# Efficacy of Selected Insecticide Sprays and Aerosols against the Common Bed Bug, *Cimex lectularius* (Hemiptera: Cimicidae)

**DOI:** 10.3390/insects7010005

**Published:** 2016-01-29

**Authors:** Changlu Wang, Narinderpal Singh, Chen Zha, Richard Cooper

**Affiliations:** Department of Entomology, Rutgers University, New Brunswick, NJ 08901, USA; singh.narinderpal@gmail.com (N.S.); cz166@scarletmail.Rutgers.edu (C.Z.); rcooper@aesop.rutgers.edu (R.C.)

**Keywords:** *Cimex lectularius*, insecticide efficacy, exposure time, substrate type

## Abstract

We evaluated the residual efficacy of four liquid sprays and four ready-to-use aerosols that are commonly used in the U.S. against a field-collected bed bug, *Cimex lectularius* L., strain with moderate resistance level to pyrethroids. The four liquid sprays were: Tandem (0.1% thiamethoxam, 0.03% lambda-cyhalothrin), Temprid SC (0.05% imidacloprid, 0.025% cyfluthrin), Transport GHP (0.05% acetamiprid, 0.06% bifenthrin), and Demand CS (0.03% lambda-cyhalothrin). The four aerosols were: Alpine (0.5% dinotefuran), Bedlam (0.4% sumithrin, 1.6% MGK 264), Bedlam Plus (0.4% sumithrin, 1% MGK 264, 0.05% imidacloprid), and Phantom (0.5% chlorfenapyr). Bed bugs were confined for 4 h to treated substrates (aged 24 h). Four substrates were tested: fabric, unpainted wood, painted wood, and vinyl. Bedlam, Demand CS, and Temprid SC resulted in ≤70% mortality on all tested substrates. Among the other five products, substrate type significantly affected their residual efficacy, except for Transport GHP, which caused ≥89.7% mortality regardless of the substrate. The effect of exposure time (5 min, 4 h, and 24 h) on the efficacy of Transport GHP and Phantom aerosol also was evaluated. A 4 h continuous exposure to Phantom aerosol or Transport GHP residue caused similar mortality to 24 h exposure and higher mortality than 5 min exposure.

## 1. Introduction

The common bed bug, *Cimex lectularius* L. (Hemiptera: Cimicidae), is an important blood-sucking insect that severely affects our quality of life. Historically, bed bugs were treated with very potent organochlorine and organophosphate insecticides in the 1940s and 1950s, which led to the subsequent disappearance of bed bug infestations in many parts of the world [1]. A resurgence of *C. lectularius* over the past 15 years has resulted in the use of many insecticide products for their control [2]. A survey of the pest control industry in the U.S. found 96% of the pest control companies used insecticide liquid sprays and 52% use insecticide aerosols for the treatment of bed bugs [3]. Similarly, consumers readily resort to insecticides in an attempt to eliminate bed bug infestations. For example, 40% and 72% of the interviewed residents in senior citizen occupied buildings in Indiana and New Jersey reported using insecticide sprays [2,4].

Because of the bed bugs’ nocturnal activity, a single insecticide application rarely exposes all bed bugs at one time. Thus high residual efficacy is necessary for killing individuals that are not exposed at the time of the application [5]. The performance of an insecticide is influenced by a variety of factors including, but not limited to, inherent toxicity and repellency of the insecticide, insect strain, developmental stage, and feeding status [6,7,8,9]. Bed bugs avoid staying on surfaces treated with deltamethrin [10], but this avoidance behavior may be affected by factors such as texture or presence of attractants [11,12]. In the tropical bed bug (*Cimex hemipterus* (F.)), another species that has also resurged in recent years, late instars are less susceptible to insecticides than younger stages and adults [6]. Non-replete *C. lectularius* are more likely to die from insecticide treatment than bed bugs with a recent blood meal [7]. It is essential to consider these factors when selecting the most effective products for use in a bed bug management program, as well as for the development and screening of insecticide products for bed bugs.

*Cimex lectularius* is cited as the most difficult urban pest to control [3]. Effective non-chemical methods exist such as steam, laundering and hot drying, vacuuming, and whole structure heat treatment [13,14]; however these methods may be labor intensive or more expensive compared to insecticide applications. Insecticides continue to be an essential component in the tool box for pest management professionals. Therefore, it is extremely beneficial to provide updated information on the performance of various insecticides developed against bed bugs. Several studies have examined the efficacy of insecticides against bed bugs using various methods under laboratory conditions. While pyrethroid spray residues resulted in good kill of a laboratory strain of *C. lectularius* [12,15], Potter *et al.* (2012) showed low efficacy of pyrethroid insecticide residues and high efficacy of dry residues of several pyrethroid + neonicotinoid insecticide mixtures against field strains [16]. In light of the prevalence of pyrethroid resistance among bed bug populations, non-pyrethroid insecticides are regarded as better alternatives [16,17,18,19,20,21]. Singh *et al.* (2014) evaluated the efficacy of essential oil and detergent products [22]. However, there are no data on the comparative efficacy of the insecticide mixtures or non-pyrethroid-based insecticide sprays. Understanding variability in efficacy of an insecticide on commonly encountered substrates is also important for selecting the most effective treatment strategies. This is critically important for assessing products in efficacy assays, particularly when data is derived for product registration. Here, we provide data on the comparative residual efficacy of three commonly used insecticide mixture products (pyrethroid + neonicotinoid) and three non-pyrethroid aerosol sprays on various substrates. A liquid pyrethroid spray and aerosol product was included for comparison.

## 2. Experimental Section

### 2.1. Bed Bugs and Experimental Conditions

A *C. lectularius* field strain (Indy) was used in this study. They were collected from multiple apartments during 2008–2009 in a building in Indiana. The strain is moderately resistant to pyrethroid insecticides. In direct spray experiments (at the rate of 4.07 mg/cm^2^ diluted solution) conducted 16 months prior to and after this study, they suffered 36% and 50% mean mortality at 5 days after direct spray with Suspend SC (0.06% deltamethrin) at the highest label rate (Bayer Environmental Science, Durham, NC, USA), indicating the resistance level of this strain was fairly consistent. They were maintained in plastic containers (4.7 cm height and 5 cm diameter; Consolidated Plastics, Stow, OH, USA) with folded construction paper (Universal Stationers Supply Co., Deerfield, IL, USA) as harborages at 26 ± 1 °C, 40% ± 10% relative humidity (RH), and a 12:12 h (L:D) photoperiod. They were fed every two weeks on defibrinated rabbit blood using a Hemotek membrane-feeding system (Discovery Workshops, Accrington, UK).

Ten nymphs (4th–5th instars) and 10 males of unknown age were placed on filter paper in each plastic dish (5.5 cm diameter and 1.5 cm height; Fisher Scientific, Pittston, PA, USA). Immediately prior to treatments dead bed bugs and bugs in the process of molting were removed and replaced. The bugs were kept in a 25 °C incubator with a photoperiod of 12:12 h (L:D). They were starved for 5–7 days prior to insecticide exposure. All experiments were conducted in a 9 m^2^ room at 26 ± 2 °C with 40%–50% RH, and a photoperiod of 12:12 h (L:D).

### 2.2. Insecticides

Four liquid spray and four aerosol spray products commonly used by professionals for controlling bed bugs in the U.S. were evaluated (Table 1). The liquid spray insecticides included one pyrethroid (Demand CS) and three neonicotinoid and pyrethroid mixtures (Tandem, Temprid SC, and Transport GHP). They were diluted with water to the rates shown in Table 1 based on label directions. Tandem is an emulsifiable concentration. Transport GHP is a wettable powder formulation. The four aerosol products included a Pyrrole (Phantom), a synergized pyrethroid (Bedlam), a neonicotinoid (Alpine), and a synergized neonicotinoid and pyrethroid mixture (Bedlam Plus). Alpine, Bedlam, and Phantom were obtained from Univar USA (Edison, NJ, USA). Other products were obtained from the manufacturer. All materials were obtained within two years period prior to this study and stored in the laboratory. Liquid sprays were diluted to the desired concentration and used same day after dilution.

**Table 1:** Insecticides evaluated in the study.

Formulation	Trade Name	Active Ingredients	Manufacturer
Capsulated Suspension	Demand CS	0.03% lambda cyhalothrin	Syngenta Crop Protection, LLC, Greensboro, NC, USA
Emulsifiable concentration	Tandem	0.1% thiamethoxam, 0.03% lambda-cyhalothrin	Syngenta Crop Protection, LLC, Greensboro, NC, USA
Suspension concentrate	Temprid SC	0.05% imidacloprid, 0.025% cyfluthrin	Bayer Crop Science LP, Research Triangle Park, NC, USA
Wettable powder	Transport GHP	0.05% acetamiprid, 0.06% bifenthrin	FMC Corporation, Philadelphia, PA, USA
Aerosol	Alpine	0.5% dinotefuron	BASF Corporation, Florham Park, NJ, USA
Aerosol	Bedlam	0.4% sumithrin, 1.6% MGK 264	McLaughlin Gormley King Company, Minneapolis, MN, USA
Aerosol	Bedlam Plus	0.4% sumithrin, 1% MGK 264, 0.05% imidacloprid	McLaughlin Gormley King Company, Minneapolis, MN, USA
Aerosol	Phantom	0.5% chlorfenapyr	BASF Corporation, Florham Park, NJ, USA

### 2.3. Substrates

Four substrates were selected to evaluate the effect of substrate type on insecticide efficacy. The substrates were: fabric (65% polyester, 35% cotton; Palencia Broadcloth, Springs Creative Products Group, LLC., Rock Hill, SC, USA), unpainted birch plywood (Revell, Inc., Elk Grove Village, IL, USA), painted birch plywood (three coats of polyurethane clear satin finish; Minwax Company, Upper Saddle River, NJ, USA), and vinyl (Armstrong World Industries Canada Ltd., Montreal, QC, Canada). Panels of each substrate in the size of 10 cm × 10 cm were prepared. The fabric was placed on brown cardboard panels.

### 2.4. Experiments

#### 2.4.1. Experiment 1. Effect of Substrate on Residual Efficacy of Insecticides

The liquid spray insecticides were applied to substrates using a Potter spray tower (Burkard Scientific Ltd., Herts, UK) at the rate of 4.07 mg/cm^2^ (1 gallon/1000 ft^2^). Each insecticide was applied to four panels of each substrate. The treated substrates were aged for approximately 24 h in a room at 26 ± 2 °C before being used. The aerosol insecticides were applied to substrates using the original insecticide containers. Control panels were sprayed with water using a trigger spray bottle (Mainstays; Walmart, Bentonville, AR). Each panel was sprayed from a distance of 15–20 cm for approximately two seconds. The mean amount of Alpine, Bedlam, Bedlam Plus, and Phantom aerosol product deposited on each fabric panel was 1.22 ± 0.08, 1.67 ± 0.10, 2.61 ± 0.23, and 0.14 ± 0.02 g, respectively, measured immediately after treatment using a Mettler Toledo PB153-5 scale (Mettler-Toledo Inc., Columbus, OH, USA). We assumed that other panels received a similar amount of insecticide per panel because the same spray method was used. The amount of Phantom deposit per panel was much lower than the other products due to the nature of this product. Phantom aerosol is oil-based and is much lighter than the other three water-based aerosol products.

The bugs were removed from the Petri dishes using a fine bristle brush and placed on the aged treated panels and confined with a 5.5 cm diameter and 1.5 cm tall plastic ring for 4 h. During this forced exposure period, bed bugs were slightly prodded using the tip of a fine brush if they formed clumps. After 4 h, the bed bugs were transferred to clean Petri dishes with a folded paper harborage. The Petri dishes were held for 14 days under laboratory conditions during which time mortality data were taken daily. A bed bug was considered dead if it could not move after being prodded gently or its legs could move slightly, but it could not crawl.

#### 2.4.2. Experiment 2. Relationship between Exposure Time and Residual Efficacy of Insecticides

Transport GHP spray and Phantom aerosol were selected for this experiment because they were the most effective liquid spray and aerosol products tested in Experiment 1. Each insecticide was applied to vinyl panels, allowed to dry for 24 h, then bed bugs were exposed to treated panels for 5 min, 4 h, and 24 h using the same methods as in Experiment 1. Each treatment combination (insecticide × exposure time) was replicated four times. In the 4 h and 24 h treatments, bed bugs were slightly prodded using the tip of a brush if they aggregated during the first four hours period. Bed bugs were transferred to clean Petri dishes at the end of each exposure period, and held for 15 days as described in Experiment 1. Mortality was recorded at 3, 7, and 15 days after exposure.

### 2.5. Statistical Analysis

Abbott’s formula was used to calculate corrected mortality [23]. Percent mortality data from each insecticide treatment were subject to analysis of variance (ANOVA) to determine the effect of substrate type, exposure time, or treatment. The data were checked for normal distribution and no transformation was needed. Means of percent mortality from various substrates, insecticides, or exposure times were separated by Tukey’s HSD test. All analyses were performed using SAS software version 9.3 [24].

## 3. Results

### 3.1. Effect of Substrate on Residual Efficacy of Insecticides

**Figure 1:**
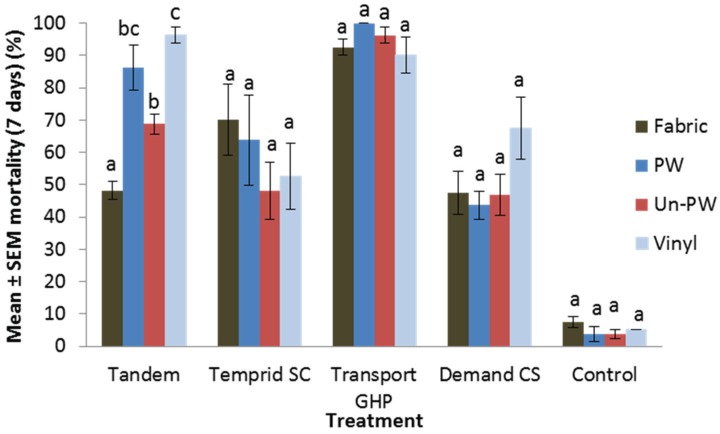
Effect of substrate type on residual efficacy of liquid sprays against *Cimex lectularius*. For each treatment, bars with the same letter are not significantly different (ANOVA, *p* > 0.05). PW: painted birch plywood; Un-PW: unpainted birch plywood.

Liquid sprays. Dead or moribund bed bugs were observed in treatments within one hour after exposure. The mortality stabilized after 7 days. Therefore, we used 7 days mortality data in the analysis. Substrate type affected the residual efficacy of Tandem (Figure 1) (*F* = 25.3; *df* = 3, 12; *p* < 0.0001), with the following significant differences: residue on fabric was less effective than on other substrates, and residue on unpainted wood was less effective than on vinyl (Tukey’s HSD test, *p* < 0.05). Substrate type had no significant impact on the efficacy of Temprid SC (*F* = 0.82; *df* = 3, 12; *p* = 0.51), Transport GHP (*F* = 1.68; *df* = 3, 12; *p* = 0.22), and Demand CS (*F* = 2.41; *df* = 3, 12; *p* = 0.12). Transport GHP caused consistently high corrected mortality (≥89.7%) on all substrates. Temprid SC and Demand CS caused ≤67.7% mortality on all substrates. Overall, both Transport GHP and Tandem were significantly more effective than Temprid SC and Demand CS; Transport GHP was significantly more effective than Tandem (Tukey’s HSD test, *p* < 0.05).

**Figure 2:**
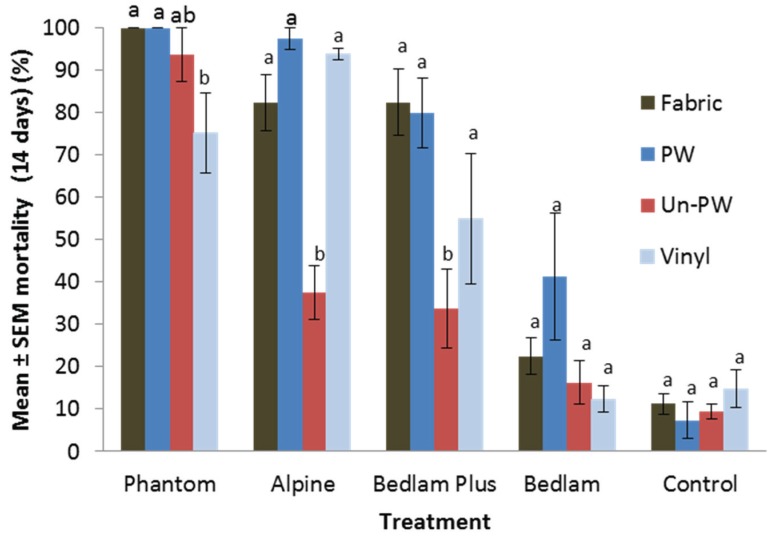
Effect of substrate type on residual efficacy of aerosol against *Cimex lectularius*. For each treatment, bars with the same letter are not significantly different (ANOVA, *p* > 0.05). PW: painted birch plywood; Un-PW: unpainted birch plywood.

Aerosol sprays. Within one hour after exposure, Alpine and Bedlam Plus treatments caused abnormal behavior of bed bugs which included, raised abdomen, curled legs, and lack of movement when touched. The mortality stabilized after 14 days, so we used 14 days mortality data in the analysis. Phantom exhibited lower efficacy on vinyl than on fabric and unpainted wood (Figure 2) (*F* = 4.3; *df* = 3, 12; *p* = 0.03). Alpine (*F* = 33.6; *df* = 3, 12; *p* < 0.0001) and Bedlam Plus (*F* = 4.7; *df* = 3, 12; *p* = 0.02) exhibited lower efficacy on unpainted wood than on other substrates. Phantom aerosol caused consistently high corrected mortality (≥70.9%) on all substrates. It was significantly more effective than other aerosol sprays on unpainted wood (Tukey’s HSD test, *p* < 0.05). Bedlam treatment caused ≤41% corrected mortality. It was significantly less effective than the other three aerosol sprays on all tested substrates (Tukey’s HSD test, *p* < 0.05). The surface type had no significant effect on its mortality (*F* = 2.4; *df* = 3, 12; *p* = 0.12).

### 3.2. Relationship between Exposure Time and Efficacy of Insecticides

**Figure 3:**
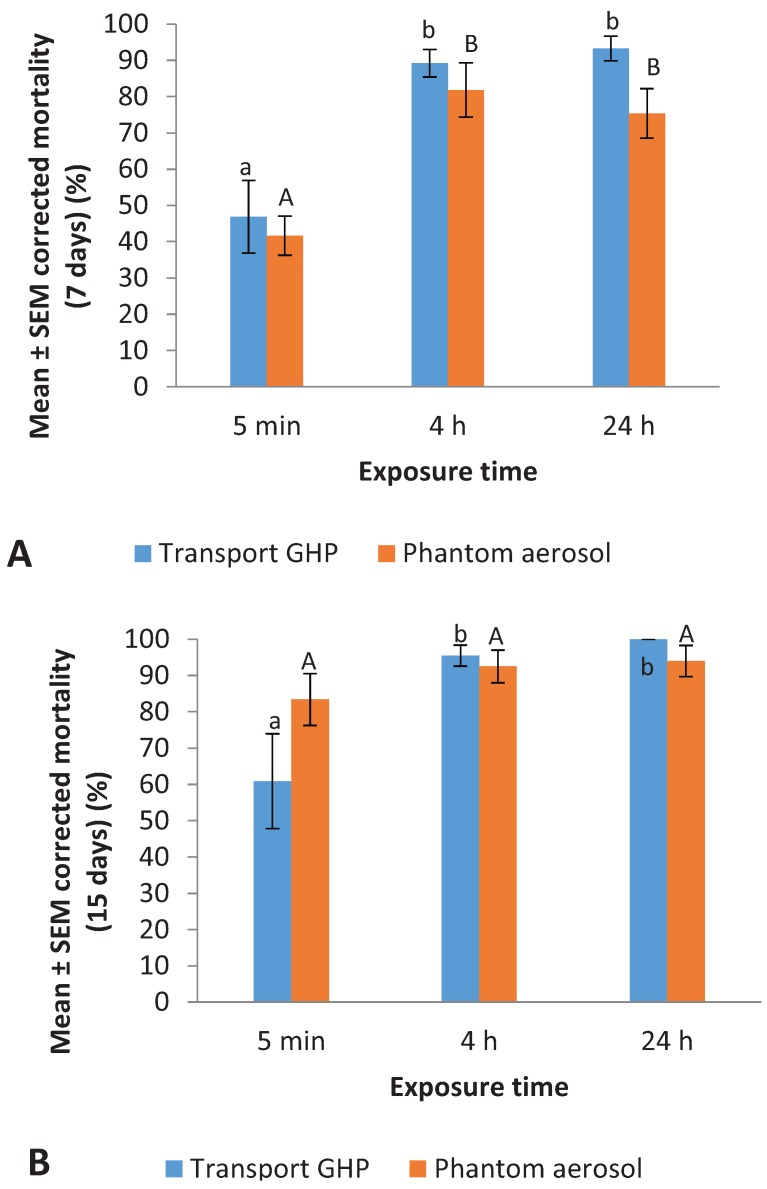
Effect of exposure time on residual efficacy of a liquid spray and an aerosol on vinyl substrate against *Cimex lectularius*. (**A**) 7 days; (**B**) 15 days. For each insecticide, bars with same letter are not statistically different (ANOVA, *p* > 0.05).

At 7 d, the mean (±SEM) mortality in the control for Transport GHP spray and Phantom aerosol was 7.4% ± 3.2% and 3.7% ± 1.2%, respectively. The 4 h and 24 h exposure resulted in significantly higher mortality than 5 min exposure for both insecticides (Figure 3A) (Transport GHP: *F* = 15.8; *df* = 2, 9; *p* = 0.001. Phantom: *F* = 10.7; *df* = 2, 9; *p* = 0.004). At 15 d, the mean (±SEM) mortality in the control for Transport GHP spray and Phantom aerosol was 16.0% ± 7.4% and 17.2% ± 4.2%, respectively. The exposure time only affected the residual efficacy of Transport GHP spray (Figure 3B) (*F* = 7.6; *df* = 2, 9; *p* = 0.01). Five minute exposures resulted in significantly lower mortality than the 4 h and 24 h exposure treatments (Tukey’s HSD test, *p* < 0.05).

## 4. Discussion

Substrate type is a major factor that impacts the residual efficacy of insecticides [25,26]. A more porous substrate generally produces lower efficacy compared to less porous substrates [27,28]. This pattern was observed for Tandem spray in this study, where its efficacy on three different substrates was: fabric < unpainted wood < vinyl. Efficacy on painted wood was similar to that on unpainted wood and vinyl. Dusts, wettable powders, suspension concentrates, or microencapsulated formulations are less likely to be affected by substrate type than emulsifiable concentrates and oil formulations [29]. This pattern was observed for Transport GHP, where the efficacy was not affected by substrate type. The performance of an aerosol formulation on various substrates was unpredictable. Efficacy of Alpine and Bedlam Plus aerosol on unpainted wood (a less porous substrate) was lower than that on fabric. Phantom aerosol on vinyl (a much less porous substrate) was less effective than that on fabric and unpainted wood. The various substrate-insecticide interaction patterns demonstrate that the performance of an insecticide on a substrate should be tested before being used.

Substrate type can be categorized into non-porous, semi-porous, and porous [26]. Bed bugs usually hide on furniture or in cracks of walls where the substrate is most likely porous. Examples of resting surfaces frequently used by bed bugs include unpainted wood, fabric, wall paper, concrete, paper, and plaster. Therefore, pesticide applications for bed bug control are typically applied to porous surfaces. A survey of pest control companies in the U.S. showed 94% respondents said their company typically treats infested beds with insecticides [3]. Of those that do treat beds, 78% treat the mattress, box spring, and frame. In light of these practices, a high residual efficacy on porous surfaces is essential when selecting insecticide sprays for bed bug control. Our study shows only Transport GHP meets this criterion. Phantom also was very effective both on fabric and unpainted wood, but lowered efficacy was observed on vinyl substrate.

Bed bugs are gregarious and tend to form clusters when not foraging [30]. A horizontal transfer effect might exist among the individuals where the active ingredient was transferred among the bed bugs [31]. Conversely, if the bed bugs were not aggregating or were active during the confinement on treated surfaces, they would have likely picked up more active ingredient through direct contact with the treated surface. We only tried to separate the clustered bed bugs during the first 4 h in Experiment 2. Additional disturbance beyond 4 h would create disturbances that might be excessive and not representing natural conditions.

Due to variations in product formula and container designs, the amount of aerosol sprays applied to each panel varied among treatments. Therefore, the different efficacy might be partially affected by the different amount deposited on each panel. For example, lower efficacy of Bedlam compared to Bedlam Plus (Figure 2) might be partially due to a lower amount of Bedlam (1.67 g/panel) applied than Bedlam Plus (2.61 g/panel). These two products contain 0.4% sumithrin and a synergist (1.6% or 1% MGK). In addition, Bedlam Plus contains 0.05% imidacloprid. How much efficacy was contributed by the addition of imidacloprid is unknown from this study. However, Phantom aerosol also was applied at much lower amount (0.14 g/panel) than other products, but was more effective on unpainted wood than other aerosol sprays, demonstrating the active ingredient and formula were deciding factors contributing to product efficacy.

A limitation of this study was that only one field strain was tested. Strain differences existed when testing residual efficacy of Temprid SC [20]. In that study, six out of nine field strains showed low mortality after continuous exposure to Temprid SC dry residue at 7 days, but eight of nine strains had high mortality response after exposure to Transport GHP dry residue. In our study, Temprid residue on various surfaces caused 48%–70% mortality to Indy strain after 7 days, indicating this strain may be representative of field strains. It would be interesting to know whether strain differences exist in their mortality response to Alpine, Bedlam Plus, and Phantom. Testing multiple strains with different insecticide exposure history will be valuable to understand the potential range of efficacy data especially when there is a concern of cross-resistance among insecticides.

In Experiment 2, the 4 h and 24 h exposure to Phantom aerosol resulted in significantly higher mortality than 5 min exposure at 7 days. By 15 days, mortality from all the exposure times became similar. Therefore, shorter exposure time will more likely reveal differences in the speed of control of an insecticide. It also may be useful for identifying subtle differences such as sub-lethal effects of insecticides.

A perceived residual efficacy of insecticides is a primary reason for pest management professionals to include them in their bed bug treatment program. Our results revealed that only Transport GHP provided high efficacy on all tested substrates. Professionals should consider the substrate type when selecting spray or aerosol products. It is advisable to incorporate other formulations or non-chemical methods in the treatment and follow integrated pest management principles to achieve satisfactory bed bug population reductions.

## 5. Conclusions

Residual efficacy of insecticide sprays varied among the four tested substrates. There was no consistent pattern between their efficacy and the porosity of the tested substrates except that Tandem exhibited lower efficacy on more porous substrates. Only one of the tested products (Transport GHP) exhibited ≥89.7% efficacy on all tested surfaces. Pyrethroids were not very effective on all tested substrates when tested against moderately resistant field strain bed bugs. A four hour exposure time was sufficient to estimate the residual efficacy of insecticide sprays.
